# Supplementary material for: Cystine/Glutamate antiporter system xc- deficiency impairs macrophage glutathione metabolism and cytokine production
Source: PLoS One. 2023 Oct 4;18(10):e0291950. doi: 10.1371/journal.pone.0291950 (PMC10550110; doi:10.1371/journal.pone.0291950)
Supplement: Attachment — Submitted filename: Response to Reviewers.pdf [file pone.0291950.s001.pdf]

Dear Reviewers,

We are grateful for the careful review and constructive comments provided by the academic editor and the reviewers concerning our manuscript entitled, "Cystine/glutamate antiporter system x_c^- deficiency impairs macrophage glutathione metabolism and cytokine production."

Below we have addressed each point of critique and outlined the modifications that we have made in the revised manuscript.

Journal requirements:

1. File naming and manuscript style have been reviewed and now align with PLOS ONE's style requirements.

2. The manuscript has undergone an intensive round of copyediting for language usage, spelling, and grammar.

3. We have expanded the Methods section to provide detailed information on (1) methods of sacrifice, (2) methods of anesthesia and/or analgesia, and (3) efforts to alleviate suffering, in line with the PLOS ONE submissions requirements.

4. Our Data Availability statement now features a DOI we have reserved where we will upload the datasets for the figures after publication.

Reviewer #1:

We appreciate the positive feedback and have made the following revisions:

- We have now included sequences in the manuscript.
- Titles have been added to align more accurately with the method's procedures.
- Product ID and any modifications or precautions done to procedures have been clarified.

Reviewer #2:

Thank you for your valuable comments. We have responded as follows:

1. We corrected the spacing issue on line 2 of the Introduction.
2. We added more references to support the statement on line 11.
3. We have made the requested citation correction on lines 23-26 and provided additional clarification on the connection between our study and the publication demonstrating the effect of Sxc deficiency.
4. A brief description of the overall findings has been added at the end of the introduction.
5. A brief description of endotoxemia and the role of LPS has been included.
6. Line 82 has been rephrased for clarity.
7. The Cas9 concentration on line 84 has been corrected.
8. We have included Figure 1 in the revised manuscript.

9. Legends for Figure 4a and 4b have been added and labels on Figure 4b have been corrected.
10. Figure 5 now includes the legend.

Reviewer #3:

Thank you for your positive feedback and for your minor requests for revision:

1. We have provided detailed information on both the ex vivo and in vivo experiments in the Materials and Methods section.
2. The phrase, "Product ID and sequences are listed in where?" has been removed from line 82.
3. The phrase, "Journal probably wants article numbers/clones of Ab used here or supplemental table." has been removed from lines 92-93.
4. We have expanded our discussion and explanation of both the ex vivo and in vivo results in the Discussion section.

We believe that these revisions have significantly improved our manuscript and look forward to your favorable response.

Yours sincerely,
Axel de Baat
